# Deep learning-aided 3D proxy-bridged region-growing framework for multi-organ segmentation

**DOI:** 10.1038/s41598-024-60668-5

**Published:** 2024-04-29

**Authors:** Zhihong Chen, Lisha Yao, Yue Liu, Xiaorui Han, Zhengze Gong, Jichao Luo, Jietong Zhao, Gang Fang

**Affiliations:** 1 Institute of Computing Science and Technology, Guangzhou University, Guangzhou, 510006 China; 2 Guangdong Provincial Key Laboratory of Artificial Intelligence in Medical Image Analysis and Application, Guangzhou, 510080 China; 3 School of Medicine, South China University of Technology, Guangzhou, 510180 China; 4 School of Information Engineering, Jiangxi College of Applied Technology, Ganzhou, 341000 China; 5 Department of Radiology, School of Medicine, Guangzhou First People’s Hospital, South China University of Technology, Guangzhou, 510180 China; 6 Information and Data Centre, School of Medicine, Guangzhou First People’s Hospital, South China University of Technology Guangdong, Guangzhou, 510180 China

**Keywords:** Multi-organ segmentation, 3D CT image, Region-growing, Deep learning, Proxy-bridging, Computational biology and bioinformatics, Engineering

## Abstract

Accurate multi-organ segmentation in 3D CT images is imperative for enhancing computer-aided diagnosis and radiotherapy planning. However, current deep learning-based methods for 3D multi-organ segmentation face challenges such as the need for labor-intensive manual pixel-level annotations and high hardware resource demands, especially regarding GPU resources. To address these issues, we propose a 3D proxy-bridged region-growing framework specifically designed for the segmentation of the liver and spleen. Specifically, a key slice is selected from each 3D volume according to the corresponding intensity histogram. Subsequently, a deep learning model is employed to pinpoint the semantic central patch on this key slice, to calculate the growing seed. To counteract the impact of noise, segmentation of the liver and spleen is conducted on superpixel images created through proxy-bridging strategy. The segmentation process is then extended to adjacent slices by applying the same methodology iteratively, culminating in the comprehensive segmentation results. Experimental results demonstrate that the proposed framework accomplishes segmentation of the liver and spleen with an average Dice Similarity Coefficient of approximately 0.93 and a Jaccard Similarity Coefficient of around 0.88. These outcomes substantiate the framework's capability to achieve performance on par with that of deep learning methods, albeit requiring less guidance information and lower GPU resources.

## Introduction

Multi-organ segmentation in 3D abdominal computed tomography (CT) images is a pivotal task for computer-aided diagnosis, radiotherapy, and surgical planning^1^. Accurate segmentation results can provide valuable information, including organ location, size, and boundary, which are crucial for clinical diagnosis and the subsequent clinical workflow^2^. However, manual annotation of organs slice-by-slice is tedious and often yields low reproducibility, attributed to the low contrast in images and the extensive number of CT slices involved^3^.

To achieve automatic multi-organ segmentation, various methods have been proposed, which can be broadly categorized into traditional segmentation methods and deep learning segmentation methods. Traditional segmentation methods include region-growing^4^, statistical shape model^5^ and structured random forest (SRF)^6^. These methods exploit inherent intensity differences to design various features^7,8^. For instance, region-growing methods use defined growing seeds and growing conditions for multi-organ segmentation^7^. However, selecting these features requires human intervention. Furthermore, since traditional segmentation methods rely on intensity values, their performance is inevitably compromised by inhomogeneous intensity and noise.

Compared to traditional methods, deep learning methods have garnered increased attention for multi-organ segmentation^2,9,10^. These methods automatically extract high-level and low-level features and encode them for segmentation. A typical segmentation network is the U-shaped network, which features an encoder-decoder structure^11^. Although they achieve great success, these methods require extensive pixel-by-pixel annotations. Moreover, as deep learning models become larger and more complex, many traditional forms of computational power provision have struggled to meet their demands^12,13^. Simultaneously, it is crucial to seriously address accompanying problems, such as overfitting^14^.

Inspired by these works, we propose an automatic 3D hierarchical framework that combines the advantages of traditional methods and deep learning to achieve multi-organ segmentation in abdominal CT images. Specifically, we first select a key slice of each 3D volume based on the intensity histogram statistics. Then, we train a supervised vision transformer (ViT) model^15^ to automatically locate the semantic central region for calculating seed points. To mitigate the effect of noise, we employ a proxy-bridged strategy that transforms the original CT images into superpixel images for multi-organ segmentation. After obtaining the segmentation results of the current slice, we calculate its centroids and use these as the growing seeds for neighboring slices, repeating the process until completion. Notably, we design three iterative termination conditions based on the morphological and density properties of the organs to ensure the iterative segmentation process terminates at the appropriate CT slice. Furthermore, the only guidance information required for the deep learning module throughout the process is the index of the semantic central patch on key slices, manually selected with a single click, thus eliminating the need for pixel-level annotations.

The main contributions of this work can be summarized as follows:

1. We propose a deep learning-aided framework for 3D abdominal multi-organ segmentation that requires only a single manual click as guidance, yet achieves segmentation performance comparable to the deep learning methods that need pixel-level annotations.
2. We propose a novel key semantic slice selection strategy that constructs an intensity histogram based on prior information and uses the intensity distribution to calculate and select key semantic slices.
3. We propose an innovative 3D proxy-bridged region-growing segmentation method that enhances image representation through proxy-bridging of input CT images, followed by segmentation leveraging the characteristics of the region-growing algorithm.
4. We propose the idea of using the region-growing algorithm to analyze 2D data for achieving 3D organ segmentation, an approach that can be generalized to other scenarios.

## Related works

### Segmentation with conventional methods

Threshold-based methods are popular techniques for organ segmentation, which subdivide the image into several cohesive regions based on the intensity of the pixels^16,17^. Numerous algorithms have been proposed in this direction over recent years, including grayscale threshold^18^, interactive pixel classification^19^, and fuzzy rule algorithms^20^. Although such methods perform well and have fast computational speed for simple tasks, they fail to take consideration of the spatial correlation information between voxels and are highly influenced by external disturbances, such as noise.

Region-growing algorithm has been well-applied in organ segmentation researches based on the high similarity in voxel grayscale intensity within the intra-organ voxels in medical images^4,7^. The growth of regions relies on the connectivity of growing seeds with adjacent voxels, which depends on predefined growth conditions or similarity criteria according to grayscale intensity or color. Statistical information and prior knowledge assimilated in algorithm to make it adaptive. The algorithm also has some limitations: (1) the segmentation results depend on the selection of growing seeds and growing conditions, which requires human intervention, (2) it works poorly for images with a large overlap of grayscale ranges, and (3) the pattern of region-growing is also sensitive to noise^21^. Therefore, region-growing is rarely used alone, many existing studies have combined it with other methods in order to achieve satisfactory performance^22,23^.

Conventional methods often exhibit limited noise resistance. Lei et al.^24^ introduced FRFCM, an enhanced fuzzy c-means (FCM) algorithm using morphological reconstruction and membership filtering to integrate local spatial information and improve anti-noise capabilities. However, it tends to overly smooth clustering outcomes, resulting in lost edge details. The RSFCM algorithm^25^ emerged as a solution to enhance image pixel relationship analysis, employing spatial correlations and a reliability indicator alongside local similarity metrics to further improve noise resistance. Most discussions surrounding noise-resistant segmentation algorithms focus on FCM, underscoring its significance. However, a major drawback exists in that the method's reliance on randomly selected initial clustering centers can detrimentally affect its efficiency if these centers are inappropriately chosen. Moreover, FCM struggles with segmentation tasks in complex scenarios, such as abdominal CT images.

### Segmentation with deep learning

Although conventional methods have been broadly utilized for segmentation tasks in biomedical imaging over the last decade, they cannot always achieve acceptable results compared to current advanced artificial intelligence (AI) techniques. Recently, deep learning has made satisfactory progress in organ segmentation^2,10^. In deep learning models, data must be organized such that the machine can clearly decipher the information. However, pixel-level annotations are very costly and time-consuming to obtain. Additionally, supervised approaches may exhibit poor performances due to over-fitting (with excessive data load) or under-fitting (with insufficient data)^26^. Therefore, deep learning methods may not always be feasible for medical image segmentation tasks, not only due to the amount of task-specific data but also the high costs associated with pixel-level annotation and the required expertise of annotators.

The high annotation cost problem can be somewhat alleviated through semi-supervised learning (SSL)^10^, wherein the model is iteratively retrained on the training set. This process involves data augmentation by adding unlabeled data and corresponding model predictions, termed pseudo-labels. In this vein, Li et al.^27^ introduced a semi-supervised approach for medical image segmentation, employing self-loop uncertainty as a novel pseudo-label to improve accuracy and efficiency with limited labeled data. MC-Net + ^28^, a novel semi-supervised network, utilizes a shared encoder and multiple decoders, enhanced by mutual consistency constraints, to refine the segmentation of indistinct regions. Recognizing the significant impact of pseudo-label quality on the model is important, as low-quality pseudo-labels may heighten the risk of judgment errors within the model.

Weakly supervised learning (WSL) is considered another solution to this problem^29^. These techniques require only weaker forms of training label annotation^30,31^, such as in the field of image segmentation, which ranges from weak to strong levels of supervision: 1) image tags, 2) size information of segmented objects, 3) points or curves labeling, and 4) bounding boxes of segmented objects. CAMEL^32^ utilized image-level labels and employed a Multiple Instance Learning method for label enrichment, enabling the automatic generation of instance- and pixel-level labels, thereby facilitating the training of segmentation models with performance comparable to that of fully supervised methods. C-CAM^33^ employed cause-effect chains to address the challenges of unclear object foreground boundaries and severe co-occurrence phenomena, generating superior pseudo masks and achieving enhanced segmentation performance. The weaker level of tagging enables models to be effectively trained, circumventing the risk of overfitting, while the information on labels that is easier to learn allows for reduced training time and lower data costs. However, WSL also presents significant challenges, including model fitting, prediction precision, and the complexities of formulating and optimizing loss functions, all of which demand considerable attention.

### Proxy-bridging strategy

Proxy-bridging results in data smoothing and feature enhancement, which can eliminate the data's individual characteristics and enhance the model's generalization ability to some extent. Different proxy types, such as edge enhancement and image smoothing, can be selected according to the research purposes.

Recently, as a typical proxy-bridged strategy, superpixels provide over-segmentation of an image by grouping pixels into homogeneous clusters based on intensity, texture, and other features. This approach represents image features with a small number of superpixels instead of a large number of pixels, significantly reducing the complexity of image post-processing^34,35^. For example,^36^ mitigates the identity mapping problem by using superpixel images as an intermediate proxy to bridge the input image and the reconstructed image.^37^ generates a salient map based on superpixel images to assist in breast lesion segmentation. Furthermore, incorporating information about the gradient change between each pixel and its adjacent ones^38^ can more effectively reveal the edge status of the tissue. Additionally, providing absolute position information assists in determining the distance between pixels, leading to more accurate and reliable segmentation^39^.

## Methods

### Overview of framework

**Figure 1:**
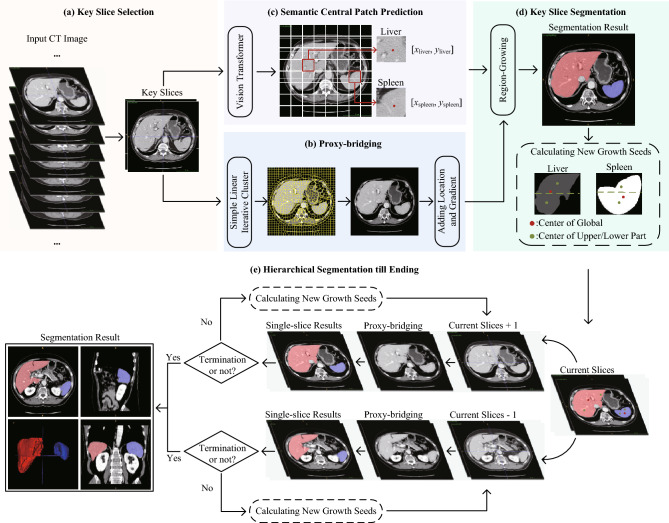
The overview of the proposed framework, which consists of five steps with different color markings: (**a**) key slice selection, (**b**) proxy-bridging images generation, (**c**) deep learning based semantic central patch prediction for key slice, (**d**) key slice segmentation, and (**e**) hierarchical segmentation.

Figure 1 illustrates the overview of the proposed framework, which consists of five steps: (1) key slice selection, (2) proxy-bridging images generation, (3) deep learning based semantic central patch prediction for key slice, (4) key slice segmentation, and (5) hierarchical segmentation. The framework utilizes manual labeling information only during the first step (key slice selection) and the third step (semantic central patch prediction). In the first step, a minimal amount of pixel-level segmentation target annotation is required for histogram analysis. Additionally, the third step required a minimal amount of supervised information for segmenting key slices, which served as prior information for propagation to both upper and lower slices. This strategy culminates in achieving end-to-end 3D segmentation without the need for extensive manual labor.

### Key slice selection

Slices with larger target organ regions (also called key slices) are easier to segment, and the confidence in the segmentation results is higher. The idea of starting segmentation from key slice and iteratively propagating the result to neighboring slices for auxiliary segmentation has been widely adopted^40,41^.

**Figure 2:**
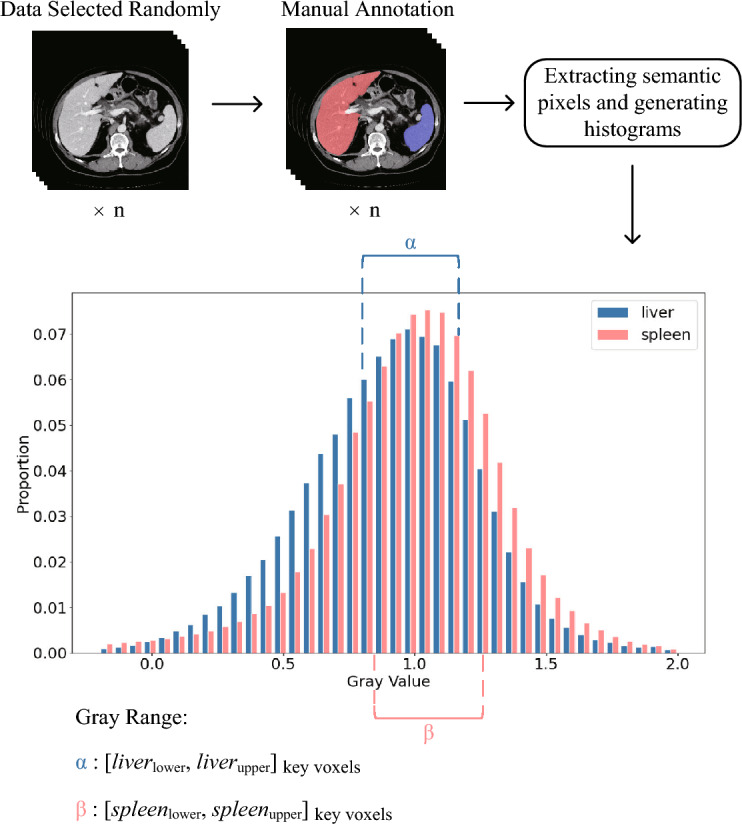
The workflow of gray histogram statistical analysis, where $n=10$ in this study, and the interval of key voxels is calculated based on the top 50% of semantic gray values.

As shown in Fig. 1a, we select the key slices of the liver and spleen in 3D CT images. The specific workflow is shown in Fig. 2. First, we randomly select 10 CT images and manually annotate the liver and spleen. We then construct histograms for the liver and spleen areas, respectively. Finally, for each histogram, we define the range of the gray values within the top 50% probabilities as a key interval, and select the slice that includes the most pixels within this key interval as the key slice.

### Proxy-bridged method

In this study, we acquire the superpixels from CT images using the SLIC algorithm^42^, which groups meaningful pixels into a superpixel by combining adjacent pixels spatially, and each superpixel is colored by the average gray value of the pixels within it.

Specifically, SLIC is a methodology based on the idea of fuzzy C-means (FCM) clustering, which requires only one parameter k, the number of superpixels expected to be derived from segmentation. Suppose the image has $N$ pixels in total, then the average size of each superpixel is $N/k$. Therefore, the distance between the centers of adjacent superpixels is approximated as $S=\sqrt{N/k}$. To avoid placing the initial center on edge or a noisy pixel, the center is moved to the lowest gradient position in its 3 × 3 neighborhood. The ensuing iterative process clusters each pixel by distance $D$, which consists of color distance ${d}_{c}$ and space distance ${d}_{s}$, and the detailed formulations are as follows:

1 $${d}_{c}=\sqrt{{\left({l}_{j}-{l}_{i}\right)}^{2}+{\left({a}_{j}-{a}_{i}\right)}^{2}+{\left({b}_{j}-{b}_{i}\right)}^{2}}$$

2 $${d}_{s}=\sqrt{{\left({x}_{j}-{x}_{i}\right)}^{2}+{\left({y}_{j}-{y}_{i}\right)}^{2}}$$

3 $$D=\sqrt{{\left(\frac{{d}_{c}}{m}\right)}^{2}+{\left(\frac{{d}_{s}}{S}\right)}^{2}}$$

where the metric of ${d}_{c}$ and ${d}_{s}$ are the L1 parametrization in the Lab color space and the coordinates in the image, respectively. In the Lab color space, the component $l$ represents luminance, and the components $a$ and $b$ represent the relative color positions ($a$: red-green, $b$: yellow-blue). For image coordinates, $x$ and $y$ denote the position of the current pixel in the 2D key slice. For aggregate distance $D$, $m$ represents the maximum color distance, controlling the compactness of the superpixels.

**Figure 3:**
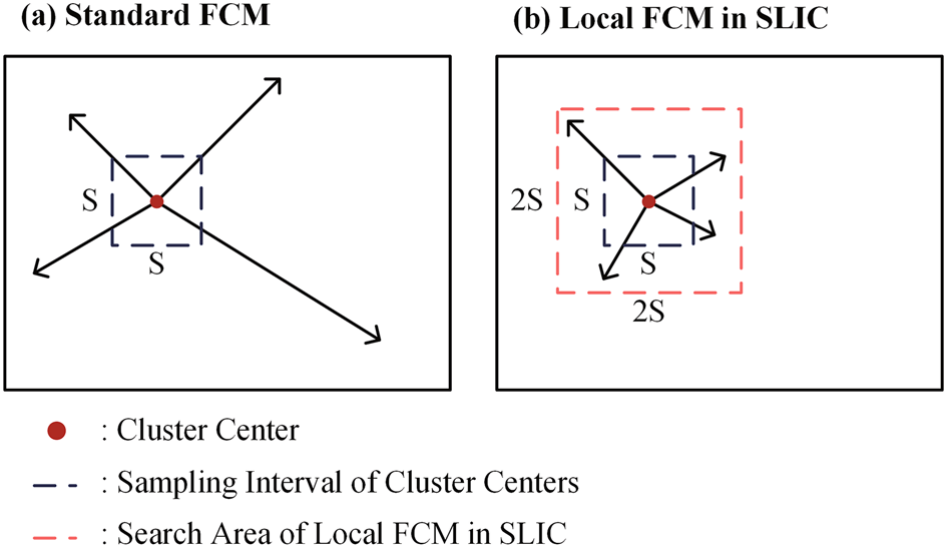
Illustrations of search areas of cluster center in standard FCM and local FCM in SLIC algorithm, in which sampling interval of cluster centers represents the average side length of square superpixels or the average distance between the centers of adjacent superpixels.

The iterative process of SLIC can be considered a type of local FCM clustering, differing from standard FCM clustering in the area of pixels searched for each cluster center. Figure 3 illustrates the search area of each cluster center in the standard FCM clustering and local FCM clustering in SLIC. The search area of each cluster center in standard FCM clustering is the whole image, which requires computing the distance from each cluster center to each pixel within the image. In SLIC, however, the search space for cluster centers is restricted to a local $2S\times 2S$ square region.

**Figure 4:**
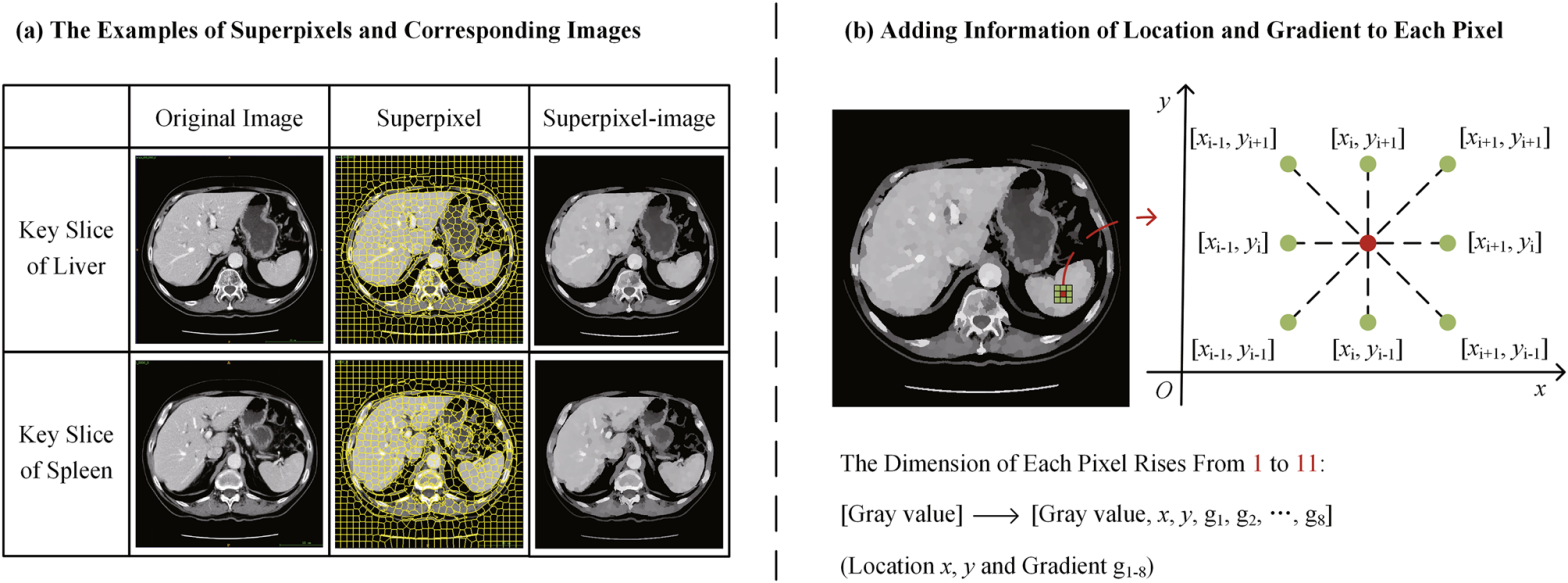
An example of proxy-bridged image. (**a**) Superpixel strategy, which the superpixel-image means the image consists of superpixels that colored with the average gray value in each superpixel. (**b**) Additional information addition, which adds the positional information of each pixel and the gradient information with its surrounding voxels to the original data.

Additionally, to facilitate better data-model fit, we integrate extra information about the position in the slice and gradient with the surrounding eight voxels. Figure 4a and b give examples of the superpixel strategy and the additional information respectively. It is noteworthy that the proxy-bridged method proposed in this study can minimize the data variability caused by the different CT scanners and CT acquisition protocols. Simultaneously, it enhances the edge information between image tissues, facilitating easier data segmentation.

### Deep learning based semantic central patch prediction

**Figure 5:**
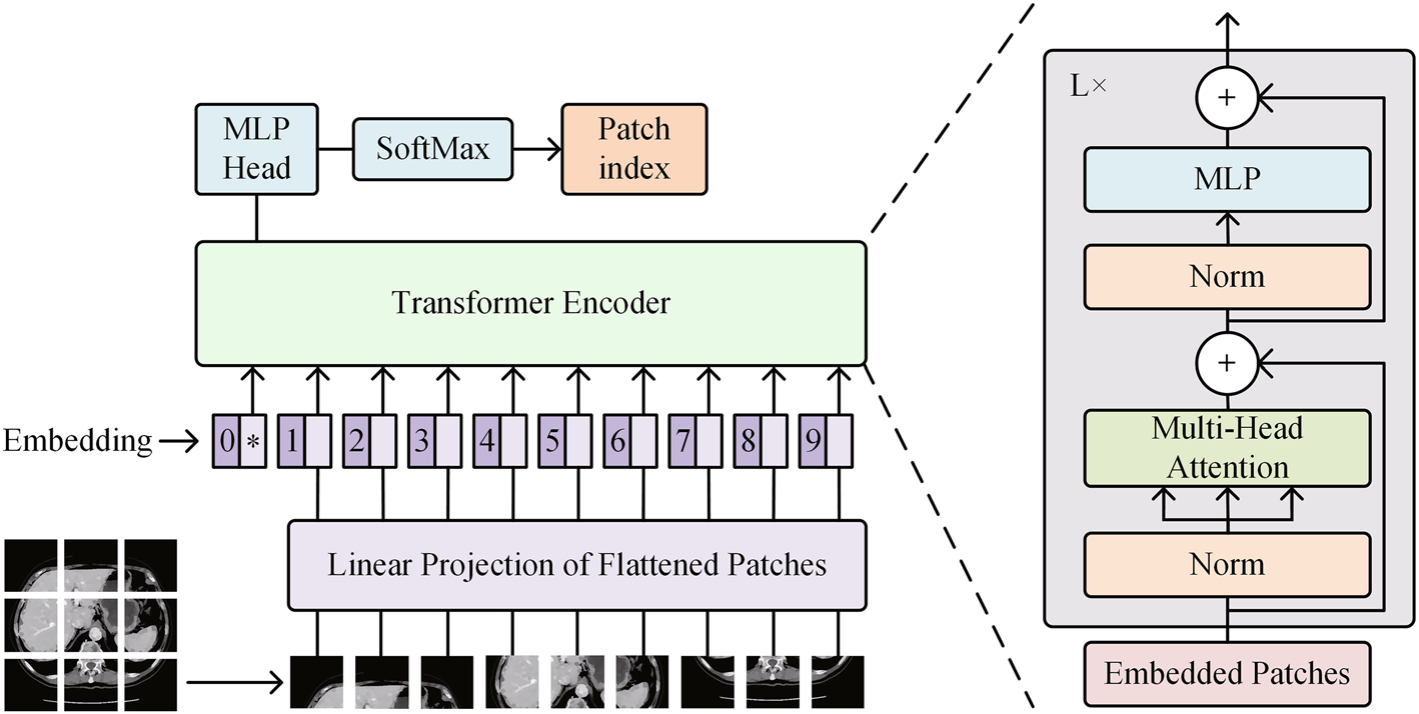
The architecture of ViT. Norm, normalization; MLP, multilayer perceptron. The slice was split into fixed-size patches, each patch was linearly embedded and added with positional information to obtain a new sequence of vectors, which was then fed to a Transformer encoder. In order to achieve the central region location, an extra learnable ‘location token’ was added to the sequence header.

To overcome the limitation of the region-growing algorithm, which requires manual selection of growing seeds, we utilize a deep learning method to automate this process and achieve adaptive segmentation. Given that the computational complexity of the Transformer is quadratic in relation to the number of tokens (i.e., sequence length), it is impractical to directly flatten the input image into a sequence for the Transformer. Therefore, as illustrated in Fig. 5, the ViT divides the image data into patches of fixed size and models their correlations as a sequence using a Transformer encoder. Additionally, the supervised signal (label) utilized in this step is the index of the patch containing the centroid. In the testing phase, once the patch containing the semantic center of the key slice has been localized, we calculate the coordinates of the initial growing seed based on the histogram statistical analysis as detailed in the ‘Key slice selection’ subsection.

Unlike existing 3D deep learning methods that require pixel-level organ annotations, the supervision signal (manual label) needed at this stage can be efficiently obtained with just a single click at the centroid of a key slice to generate the patch index. Furthermore, these key slices can be automatically identified using the ‘Key slice selection’ strategy. This implies that the time required to annotate a single conventional 3D CT image of an organ (e.g., liver) at the pixel level could, in this step, be leveraged to generate supervision signals for hundreds of CT images.

Assume that the input image is $x\in {\mathbb{R}}^{H\times W}$, it is reshaped into a sequence of flattened patches ${x}_{p}\in {\mathbb{R}}^{N\times {P}^{2}}$. Here, $H\times W$ denotes the shape of original image, ${P}^{2}$ represents the size of each split patch, and $N=HW/{P}^{2}$ indicates the total number of patches. Initially, patch embeddings ${E}_{patch}$ are obtained by projecting ${x}_{p}$ to $D$ dimensions using a trainable linear projection. Subsequently, position embeddings ${E}_{pos}$ are added to the patch embeddings to preserve positional information. It is noteworthy that an additional learnable ‘location token’ (${Z}_{0}^{0}={x}_{loc}$) is introduced to pinpoint the central region location, with the state at the output of the Transformer encoder (${Z}_{0}^{L}$) serving as the regional representation. The detailed calculation of embeddings is specified as follows:

4 $${Z}_{0}=\left[{x}_{loc};{x}_{p}^{1}E;{x}_{p}^{2}E;\dots ;{x}_{p}^{N}E\right]+{E}_{pos},E\in {\mathbb{R}}^{{P}^{2}\times D},{E}_{pos}\in {\mathbb{R}}^{\left(N+1\right)\times D}$$

And then, ${Z}_{0}$ is fed into Transformer encoder^43^, comprising $L$ layers that each consist of multi-head self-Attention (MSA) and multi-layer perceptron (MLP) blocks. Layer normalization (LN) is applied before each block, and residual connections are implemented after each one^44,45^. This process can be described by the following formulas:

5 $${Z}_{l}{\prime}=MSA\left(LN\left({Z}_{l-1}\right)\right)+{Z}_{l-1},l=\mathrm{1,2},\dots ,L$$

6 $${Z}_{l}=MLP\left(LN\left({Z}_{l}{\prime}\right)\right)+{Z}_{l}{\prime},l=\mathrm{1,2},\dots ,L$$

7 $$y=LN\left({Z}_{l}^{0}\right)$$

After the Transformer Encoder, the output $y$ is reshaped to a size of $\left(1, {N}_{region}\right)$ and subsequently subjected to a softmax activation function to determine the index of the central region. The network is trained using the classical cross-entropy loss ${L}_{CE}$, as defined in Eq. (8):

8 $${L}_{CE}=\frac{1}{N}\sum_{i=1}^{{N}_{region}}{Y}_{i}log{y}_{i}+\left(1-{Y}_{i}\right)log\left(1-{y}_{i}\right)$$

where ${Y}_{i}$ represents whether the i-th patch corresponds to the central region (e.g., 1 yes, 0 no). Finally, by leveraging key interval, the key voxels within the identified central region are determined. The coordinates of these voxels are then averaged to calculate the growing seed for the subsequent region-growing step.

### Hierarchical region-growing segmentation

We propose a hierarchical segmentation strategy for multi-organ segmentation, as shown in Fig. 1 (d) and (e). Initially, we combine the proxy-bridged key slice with a growing seed for segmentation. Subsequently, we initialize two lists ${L}_{global}$ and ${L}_{local}$ to store global seeds and local seeds, aimed at segmenting adjacent slices. As shown in Fig. 1d, global seeds represent the centers of the organ across all slices, while local seeds correspond to three centers (global, upper, and lower parts) in the current slice. Introducing local seeds effectively reduces the risk of incomplete segmentation that occurs when the global semantic center is located at noisy voxels, such as vessels or lesions. During the region-growing segmentation, ${L}_{global}$ and ${L}_{local}$ are employed to calculate the mean and variance, respectively, determining the gray value interval for segmenting each slice. In this way, in terms of the segmentation interval in each slice, the global seeds can be used to ensure the medial axis, and the local seeds information can be used to calculate the upper and lower limits. Finally, we continue the process until the end of segmentation. To be mentioned, the segmentation results of each slice are performed a 2D closing operation to ensure the integrity of the semantic parts, and a 3D closing operation at the end of the hierarchical segmentation to ensure the coherence and consistency of semantic parts between the slices. This slice-by-slice parameter determination approach exhibits adaptive control and generalization capabilities, effectively mitigating over- and under-segmentation issues caused by disparate categorical assignments of identical intensity levels across different samples or slices. Additionally, when over- or under-segmentation occurs in a particular layer, its impact on the region-growing of the subsequent slice is relatively minor.

**Figure 6:**
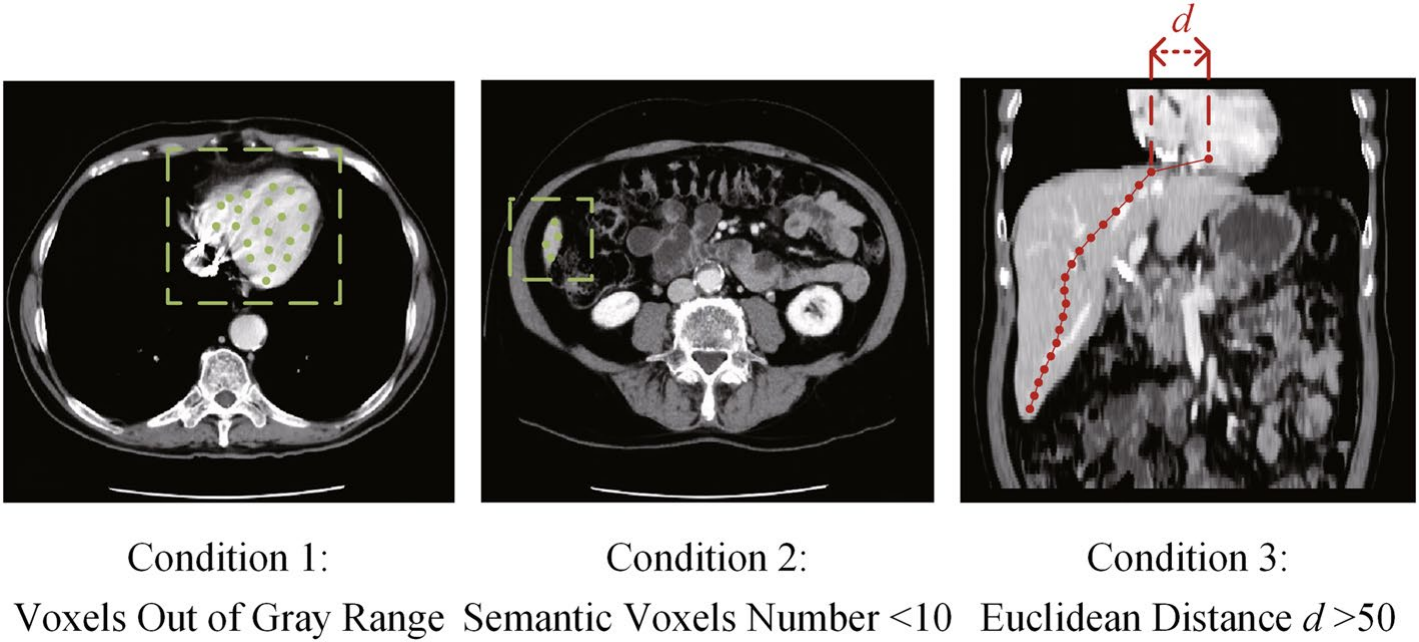
Iteration termination conditions in hierarchical segmentation, where the iteration ceases when one of them is satisfied. The gray range in condition 1 is obtained by histogram analysis in Fig. 2.

To decide whether to continue with the hierarchical iterative segmentation, we have experimentally defined three termination conditions, as illustrated in Fig. 6: (1) All growing seeds of the current slice fall outside of the key voxel gray scale range, (2) The number of semantic voxels resulting from the current slice’s segmentation falls below 10, and (3) The global semantic center of the current slice is excessively distant from the adjacent 10 layers, with a maximum Euclidean distance greater than 50 units between the global centers of each slice.

The comprehensive algorithm for the Hierarchical Region-growing Segmentation Algorithm is outlined in Algorithm 1.

**Algorithm 1:**
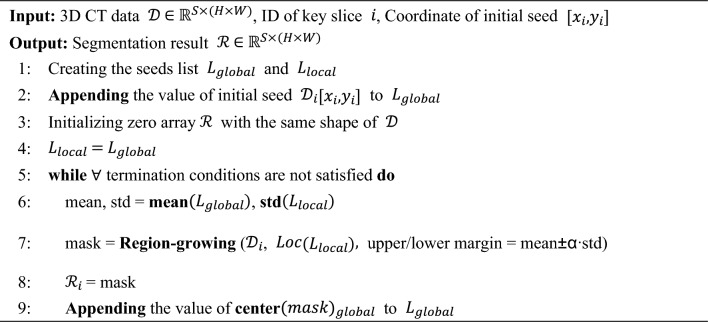
Hierarchical region growing segmentation algorithm

### Evaluation metrics

Two metrics were introduced to evaluate the properties of the growing seed localization model in this paper, accuracy (ACC) and error Euclidean distance (EED). Where ACC denotes the model's success rate in localizing the growing seed's region, whereas EED measures the distance between the localized region and the ground truth when localization at the patch level is incorrect (e.g., edge-adjacent is 1, vertex-adjacent is $\sqrt{1+1}=\sqrt{2}$, with the measurement unit being the patch).

Furthermore, to assess the segmentation performance of the proposed framework, six evaluation metrics were employed in this study. The Dice Similarity Coefficient (DSC) assesses the accuracy of model segmentation, calculated as the intersection of two masks divided by the total area of both masks, with Eq. (9) detailing this calculation. The Eq. (10) represents the Jaccard Similarity Coefficient (JSC)^46^, which imposes stricter penalties on over- and under-segmentation compared to the DSC.

9 $$DSC=\frac{2TP}{FP+2TP+FN}$$

10 $$Jaccard=\frac{TP}{FP+TP+FN}$$

where TP (True Positive) and FP (False Positive) represent semantic voxels that are correctly and incorrectly classified, respectively. Similarly, TN (True Negative) denotes correctly classified background voxels, while FN (False Negative) indicates background voxels that are incorrectly classified.

Three additional metrics recall, specificity, and precision are routinely adopted to evaluate the segmented result. Recall, also known as sensitivity, focuses on the model's ability to detect true positives. Specificity, also referred to as the true negative rate, measures the proportion of background voxels that are correctly segmented. These three metrics are formulated as Eqs. 11, 12, and 13, respectively.

11 $$Recall=\frac{TP}{TP+FN}$$

12 $$Specificity=\frac{TN}{TN+FP}$$

13 $$Precision=\frac{TP}{TP+FP}$$

The above metrics are focus on the internal voxel composition of the segmented mask, and in order to evaluate the model more comprehensively, this study incorporates Hausdorff Distance 95 (HD95) for a more holistic evaluation, specifically to assess boundary similarity with the ground truth. Defined in Eq. (14), HD95 calculates the minimum distance for each voxel in set $x (or y)$ to set $y (or x)$, subsequently adjusting the maximum of these distances by 95% to mitigate the effect of outliers^47^.

14 $$HD95={max}_{95\%}\left\{\genfrac{}{}{0pt}{}{max}{x\in X}\genfrac{}{}{0pt}{}{min}{y\in Y}d\left(x,y\right),\genfrac{}{}{0pt}{}{max}{y\in Y}\genfrac{}{}{0pt}{}{min}{x\in X}d\left(x,y\right)\right\}$$

## Results

### Dataset and Preprocessing

In this study, the proposed method was validated on a mixed dataset^48^, which collected from MSD^49^, NIH^50^, KiTS^51^, and LiTS^52^. 330 contrasted CT volumes with complete annotations of liver and spleen were selected, the resolution of all CT volumes is 512 × 512 and the slice thickness ranges from 1.25 to 5 mm. All of them were rescaled within the range of [-240, 360] and normalized to zero mean and unit variance. In addition, a median filter with neighborhood size of 3 × 3 is used for spatial smoothing.

During the ‘semantic central patch prediction’, for the training of the ViT model, data were allocated into training, testing, and validation sets with the ratio of 9:1:1 (270: 30: 30). To better harness the value of the data and increase the quantity, data augmentation was performed by selecting three of the key slices, instead of only the single key slice, from each dataset under the ‘Key Slice Selection’ strategy. Consequently, a total of 990 slices (810: 90: 90) were utilized for the training of the localization model.

### Implementation details

During the deep learning model training in the subsection ‘Deep learning based semantic central patch prediction’ in ‘Methods’, the optimizer is the Adam optimizer^53^, the training epoch is set as 500, the learning rate is set as 0.001, and the others are the default settings of the native ViT. The scale of each slice in both the initial inputs 3D CT data and the proxy image is 512 × 512, and the size of patches in ViT is 32 × 32. In the segmentation of the liver and spleen, the simple linear iterative clustering (SLIC) algorithm generates 800 and 1300 superpixels, respectively. The hyperparameter $\alpha$ in Algorithm 1 is adjusted to 3, dictating the degree of interval amplification.

### Segmentation performance

#### Growing seed localization

**Figure 7:**
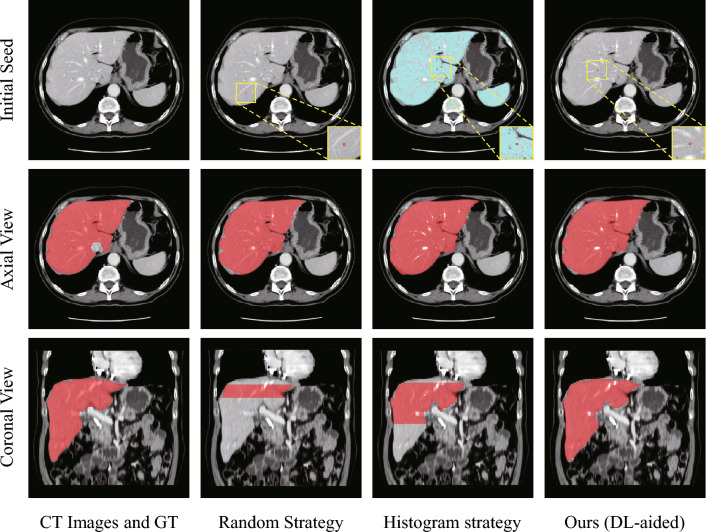
Seed localization and segmentation results in the liver using different methods. The random strategy involves multiple selections at random until a liver voxel is hit, while in the histogram strategy, the blue voxels represent the key liver voxels obtained. The red dots in the first row indicate the positions of the initial seeds. The axial view results in the second row show the segmentation results of the key slices, and the coronal view in the third row illustrates the stopping positions of iterative segmentation.

Noting that after proxy-bridging, region-growing from any voxel inside the semantic scope can achieve satisfactory segmentation results in single key slice. Therefore, as shown in Fig. 7, to further articulate the necessity of employing a deep learning-aided method that involves GPU resources in this step, we compared it with both a random strategy and a histogram-based localization strategy. In the random strategy, due to the difficulty of successfully randomizing the growth seed onto the segmentation target in a single attempt for key slice (with an average success rate of 10.32% for the liver and 2.21% for the spleen), the experiment continuously generates random seeds until one lands on a liver voxel before initiating the experiment. In the histogram-based localization strategy, the initial growth seed is determined by calculating the centroid of the key voxels selected through histogram analysis on the current key slice. Due to the overlapping key intervals of CT values for liver and spleen voxels, and the substantially larger size of the liver compared to the spleen, it is challenging to select growth seeds for the spleen using this strategy. Therefore, the experiment primarily focuses on the analysis of liver segmentation.

It can be observed that the growing seed obtained through the deep learning-aided method not only play a crucial role in segmenting key slice but also in controlling the termination of iterative segmentation. This is attributed to the fact that, as indicated by the third condition in Fig. 6, the termination of the subsequent iterative slice-by-slice segmentation is contingent upon the coherence of the growing seeds in each slice, and the selection of seeds situated too far from the semantic center may precipitate the premature and abnormal termination of the iterative process. Furthermore, within the improved region-growing algorithm that follows, the semantic center voxel of each layer collaboratively establishes the medial axis of the gray value interval. An unstable selection strategy for growth seeds may result in significant fluctuations within the gray value interval, thus adversely affecting segmentation efficacy.

To evaluate the performance of the growing seed localization model in this study, we compared it with two others common models (VGG16^54^ and ResNet50^55^), and these models are evaluated using 90 slices (3 slices are selected for each testing 3D CT data). Table 1 displays the average values of ACC and ED of the three models. The model used in this study demonstrates superior performance compared to the other two, achieving an ACC of over 0.93 and an EED is merely 1.1391 patches.

**Table 1:** The evaluation metrics (ACC and EED) results of three growing seed localization models, the improved ViT used in this study achieved the optimal performance (bold shown).

	ACC	EED
VGG16	0.8222	1.2803
ResNet50	0.8556	1.1725
This Study (ViT)	**0.9333**	**1.1391**

#### Comparison with other segmentation methods

In this study, as the proposed segmentation framework combines traditional statistical methods with deep learning, we compare the segmentation performance of the proposed method with four typical segmentation methods: SRF^6^, 3D-UNet (3DU)^11^, 3D-Attention UNet (AttnUNet)^56^, and UNETR^57^. SRF utilizes ensemble learning to segment volumetric data effectively. 3DU is known for its strong generalization ability and simple structure. AttnUNet emphasizes important features using attention mechanisms, enhancing its performance in complex tasks. UNETR, a pure Transformer architecture, handles diverse datasets without traditional convolutional layers.

Tables 2 and 3 list the quantitative results of liver and spleen, respectively. From the experimental results, the performance of the proposed framework in this study can be compared to deep learning methods and much higher than traditional method (SRF) overall. In liver segmentation, the proposed framework underperforms compared to AttnUNet and 3DU in terms of DSC, JSC, and Recall, yet it surpasses UNETR. This indicates that for liver with significant adhesion to surrounding tissues, the framework can more accurately differentiate liver voxels from other voxels, achieving a level of performance comparable to deep learning methods in this task. For the spleen, which is comparatively more autonomous, of a more regular shape, and exhibits lesser adhesion to surrounding tissues, the proposed framework outperforms in five metrics (except Recall). This demonstrates that for targets that are both more regularly shaped and more spatially independent, the framework enhances the efficacy of region-growing algorithms. By analyzing voxel intensities and their relative differences across layers, it achieves a finer distinction between the target and the background, thereby securing superior outcomes. In addition, it is obvious that the framework has superior potency in the HD95 (8.24 in liver and 2.26 in spleen), thanks to the control of segmentation by connectivity and gray scale interval in hierarchical segmentation.

**Table 2:** The segmentation performance of three different methods based on liver.

	DSC	JSC	Recall	Specificity	Precision	HD95
SRF	0.7820	0.6420	0.7325	0.9915	0.8387	15.4271
3DU	0.9405	0.8876	0.9712	0.9954	0.9117	9.7741
AttnUNet	0.9441	0.8962	0.9806	0.9957	0.9160	9.5898
UNETR	0.9209	0.8398	0.9389	0.9937	0.8980	13.7268
Ours	0.9343	0.8772	0.9513	0.9966	0.9192	8.2401

**Table 3:** The segmentation performance of three different methods based on spleen.

	DSC	JSC	Recall	Specificity	Precision	HD95
SRF	0.7978	0.6636	0.7665	0.9989	0.8319	8.7047
3DU	0.9260	0.8623	0.9195	0.9995	0.9327	4.9305
AttnUNet	0.9313	0.8755	0.9153	0.9996	0.9514	4.1720
UNETR	0.9281	0.8691	0.9063	0.9992	0.9407	7.7884
Ours	0.9359	0.8800	0.9111	0.9998	0.9635	2.2608

**Figure 8:**
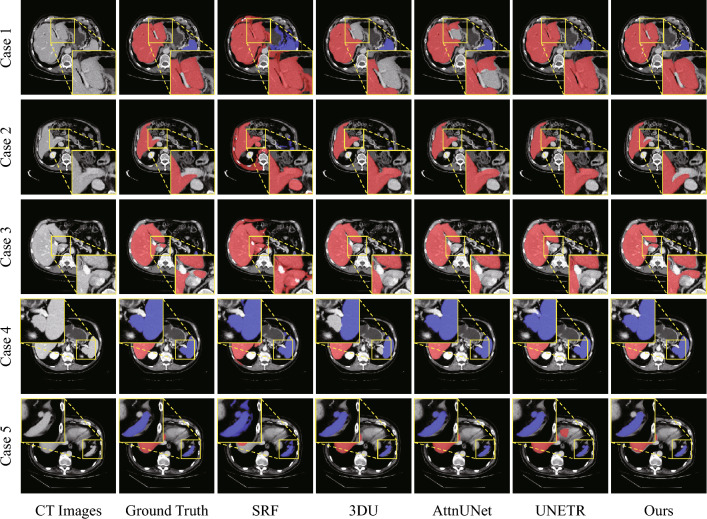
Visualization of the segmentation results of the proposed method and four other comparative methods, including SRF, 3DU, AttnUNet, and UNETR. The enhanced focus area signifies the effectiveness region of the proposed method, with Cases 1–3 primarily discussing liver segmentation, while Cases 4–5 are devoted to spleen segmentation.

Figure 8 shows the visualization of segmentation results of these methods, with cases 1–3 primarily focusing on the segmentation of the liver, and cases 4–5 on the segmentation of the spleen. Overall, the segmentation performance of the proposed framework significantly surpasses that of the traditional method SRF, and it demonstrates superior capabilities in handling specific scenarios compared to deep learning methods. Specifically, case 1 is characterized by a close adhesion between the liver and the stomach, with similar CT values, where 3DU and AttnUNet failed to accurately detect and segment the liver. UNETR, on the other hand, mistakenly included the intervening venous vessels as part of the segmentation target. The proposed framework, however, is capable of accurately and completely segmenting the liver through voxel intensity and connectivity analysis. Case 2 presents a similar situation, where all comparative methods mistakenly identified adjacent venous vessels as part of the liver, with only the proposed method being able to accurately discern it. Case 3 exhibits a small protrusion of liver tissue near the aorta that the three deep learning comparison methods failed to detect and segment. Although SRF managed to segment this area, it also resulted in severe over-segmentation. The proposed method, however, successfully segmented this area through connectivity and intensity assessments based on region growing. For case 4, 3DU showed under-segmentation of the spleen, while UNETR incorrectly included a portion of the splenic vein in its segmentation target. The proposed framework displayed stable performance, achieving segmentation results on par with AttnUNet. In case 5, there is a lymph node adjacent to the spleen with similar CT values, which all comparison methods misidentified as part of the spleen. However, the proposed method was able to exclude it, achieving a more precise segmentation.

#### Statistical analysis on significance

To determine the significance of segmentation results and reveal genuine differences between the proposed and comparative methods, we employed Student's t-tests to compute p-values for both DSC and HD95 metrics, as depicted in Table 4. DSC indicates the overlap between segmentation results and ground truth, while HD95 reflects the consistency of segmentation results with boundaries of ground truth when compared to other methods. It is observed that compared to all comparative methods, we consistently achieve *p* < 0.05, indicating a statistically significant difference with our approach.

**Table 4:** P-values for statistical analysis between all comparative methods and proposed method.

Method	Liver		Spleen	
	DSC	HD95	DSC	HD95
SRF	5.21 × 10^–34^	2.46 × 10^–5^	1.22 × 10^–26^	4.82 × 10^–8^
3DU	1.06 × 10^–6^	2.75 × 10^–6^	1.57 × 10^–2^	1.49 × 10^–10^
AttnUNet	2.37 × 10^–21^	4.62 × 10^–4^	2.90 × 10^–2^	4.23 × 10^–16^
UNETR	9.20 × 10^–6^	2.80 × 10^–6^	1.30 × 10^–3^	5.59 × 10^–16^

#### Computational resource consumption analysis

The consumption of computational resources also serves as a standard for gauging model performance. In both academic and industrial settings, minimizing computational resource use without sacrificing model efficacy is crucial. Noting that the SRF model, along with the iterative region-growing segmentation within our framework, does not involve GPU parallel computing nor does it incurs GPU memory consumption. Consequently, the SRF model is not considered in the calculation of resource consumption, and the discussion regarding the proposed framework is centered on the 2D ViT aspect. Table 5 compares the resource consumption of the proposed framework with that of three deep learning methods, focusing on parameter count (PARAMs) and floating-point operations per second (FLOPs).

**Table 5:** Comparison results of required computational resources in liver segmentation, the proposed framework has the lowest required FLOPs and PARAMs (bold shown).

Method	FLOPs (G)	PARAMs (M)
3DU	138.94	38.12
AttnUNet	602.39	47.01
UNETR	770.65	111.95
**Ours (2D ViT)**	**2.04**	**8.68**

It can be observed that the FLOPs required by the proposed framework amount to only about 1/68 of those for 3DU and approximately 1/378 of those required by UNETR, which similarly employs the Transformer architecture. Simultaneously, when compared to CNN-based models, the PARAMs needed by this framework amount to merely 22.77% for 3DU, 18.46% for AttnUNet, and just 7.75% for the Transformer-based architecture UNETR. This is attributed to deep learning methods requiring the analysis and judgment of every voxel within 3D feature maps, while this framework only needs to locate the Patch index of the semantic center in key slices. Consequently, with the premise of ensuring end-to-end 3D segmentation performance, the resource consumption required by this framework is significantly lower than that of existing deep learning methods.

### Ablation studies

A series of ablation experiments were conducted to validate the effectiveness of each component within the framework, with Table 6 reporting the results (DCS metric) of these ablation experiments. (a) The effectiveness of proxy-bridging method: This involved introducing (1) a superpixel image (SI) and (2) adding gradient and positional information (GPI) to each pixel, enhancing data representation and smoothing. From exp. 1–3, we can observe that the segmentation framework performs better with proxy-bridging than without, which demonstrates that the introduction of SI can eliminate the noises, and GPI can further strengthen the boundaries to achieve better segmentation performance to a certain extent. (b) The impact of the number of superpixels: This number influences the size and the boundary pattern of each aggregation cluster. The results of exp. 4–7 show that too much or too few numbers of superpixels cannot achieve the optimal segmentation performance, and 1300 is the preferred parameter in this study. (c) The performance of the proposed termination conditions (as shown in Fig. 6): Exp. 8–11 indicate that all three conditions enhance segmentation performance, with their combination yielding the best results. (d) The effectiveness of morphological closing operation (post-processing method): The results of exp 12–14 demonstrate that closing operation could be an effective tool for correcting the segmentation results and getting better segmentation. (e) The necessity of local seeds in slice-wise segmentation: Exp. 15–17 demonstrate how introducing local seeds, based on global seeds, influences segmentation results. Here, ‘2-local seeds’ refers to the center voxels of the upper and lower parts, while ‘4-local seeds’ refers to the center voxels of the top-left, top-right, bottom-left, and bottom-right parts. Results show that the selection of ‘2-local seeds’ performs better than ‘4-local seeds’, which mainly because too many local seeds may force the framework to focus on a small portion of the region near the boundary and lead to incorrect segmentation results.

**Table 6:** The DSC results of different conditions for segmentation of liver, with the conditions under which optimal DSC can be achieved in each setup shown in bold.

Exp	Different conditions	DSC
1	Without proxy	0.9061
2	SI	0.9311
3	**SI + GPI**	**0.9343**
4	500 superpixels	0.9126
5	900 superpixels	0.9217
6	**1300 superpixels**	**0.9343**
7	1700 superpixels	0.9163
8	Without termination conditions	0.5614
9	Condition 1	0.6496
10	Condition 1, 2	0.7817
11	**Condition 1, 2, 3**	**0.9343**
12	Without closing operation	0,8741
13	2D closing operation	0.9117
14	**2D, 3D closing operation**	**0.9343**
15	Without local seeds	0.8577
16	**2-local seeds**	**0.9343**
17	4-local seeds	0.8814

## Discussion

With the advancement of deep learning, there are increasingly more researches to conduct medical-industrial combination to solve tasks in medical image field. Combining emerging technologies to offset the limitations of traditional methods has become particularly meaningful, enhancing the analysis and processing capabilities for radiographic images. This study integrates deep learning, the proxy-bridging concept, and an improved region-growing algorithm to develop a novel hierarchical segmentation framework, aiming to match the performance of advanced deep learning models. To evaluate the effectiveness of the proposed method, we compared it with four other methods. The experimental results indicate that the proposed method could achieve great performance on liver and spleen segmentation.

Furthermore, the ablation studies reveal that: (1) proxy-bridging technique that combines superpixel images as well as additional gradient and positional information can improve the fitness of the framework model to the segmentation task; (2) for vision tasks with hierarchical segmentation ideas, utilizing morphological closing operation for post-processing can considerably improve segmentation performance; (3) in terms of the region-growing algorithm, not only the location of the initial growing seeds need to be concerned, but the number of them also has a considerable impact on the segmentation performance. Therefore the determination of a specific number of seeds by a specific method for a specific task is also important.

**Figure 9:**
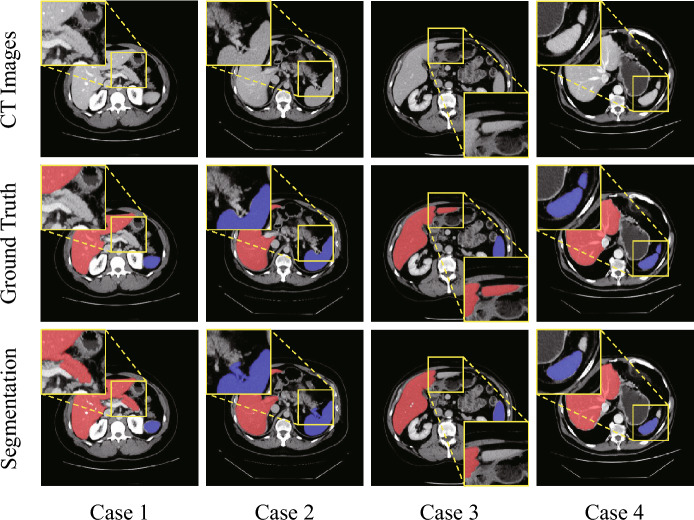
Examples of cases with poor segmentation, with the magnified areas indicating the portions of segmentation errors. Cases 1 and 2 exhibit over-segmentation attributed to adhesion of tissues and low contrast, whereas Cases 3 and 4 present under-segmentation due to the discontinuity of the targeted segmentation regions.

Figure 9 presents four cases with poor segmentation performance. In the initial pair of scenarios, the segmentation targets are strongly adhered to adjacent tissues with poor contrast, resulting in over-segmentation of hepatic tissue in Case 1 and splenic tissue in Case 2. The subsequent pair of cases reveal segmentation targets that are not contiguous within the imaging slice. Both the liver in Case 3 and the spleen in Case 4 suffer from the intrinsic limitations of the region-growing algorithm, which is ineffective at segmenting isolated outlier regions. These cases elucidate two significant limitations of the proposed framework: (1) it is difficult to segment voxels that are strongly adhered to irrelevant tissues and have extremely similar gray intensity, wherein the framework may treat these adhered voxels as entire and segment them; (2) due to the nature of region-growing, it has high requirements for the connectivity of segmented organ, and the non-connected organ cannot be completely segmented once time.

## Conclusions

In this study, we propose a deep learning-aided 3D proxy-bridged region-growing framework designed for multi-organ segmentation. Specifically, the framework initially selects the key slice based on statistical information, setting the stage for proxy-bridging. It then identifies the semantic central patch based on deep learning methods and calculates growing seed. Subsequently segments it using region-growing algorithm, and finally iteratively segments the neighboring slices based on the segmentation result until completion. Experimental results demonstrate that the framework achieves satisfactory segmentation of the liver and spleen, with a DSC over 93%, a JSC around 88%, and significant improvements in HD95. In conclusion, this framework demonstrates performance comparable to various deep learning methods but requires fewer GPU resources and relies solely on a single-click for supervision (label) in the key slice, eliminating the need for pixel-level annotations. As a universal method, this framework can be generalized to other scenarios.

## Data Availability

The datasets analyzed during the current study are available in the AbdomenCT-1 K repository, https://github.com/JunMa11/AbdomenCT-1K.
